# 1213. Application of Short Course Criteria for Community-Acquired Pneumonia (CAP) Among Patients Admitted Within a 17-community Hospital Telehealth Antimicrobial Stewardship Program (Tele-ASP)

**DOI:** 10.1093/ofid/ofad500.1053

**Published:** 2023-11-27

**Authors:** Aunalia Rasmussen, Stephanie Shealy May, Stephanie Shealy May, John J Veillette, Todd J Vento, Brandon J Webb

**Affiliations:** Intermountain Health, Murray, Utah; Intermountain Health, Murray, Utah; Intermountain Health, Murray, Utah; Intermountain Healthcare, Murray, UT; Intermountain Healthcare, Murray, UT; Intermountain Healthcare, Murray, UT

## Abstract

**Background:**

A recent randomized controlled trial by Dinh et al. found three days of antibiotics was appropriate for certain community-acquired pneumonia (CAP) patients, but the real-world applicability of this study remains to be evaluated. In February 2022, a telehealth antimicrobial stewardship program (Tele-ASP) intervention was implemented across 17 small community hospitals to identify patients meeting clinical stability criteria used by Dinh et al. for short course therapy (3 days). An alert was implemented to notify the infectious diseases telehealth (Tele-ID) pharmacist of patients on room air for whom an antibiotic was ordered for pneumonia.

**Methods:**

This IRB-approved study aimed to evaluate feasibility and impact of the intervention. Patients with severe or complicated CAP, known immunosuppression, or who were transferred were excluded. The primary outcome was total antibiotic duration. Secondary outcomes included whether the total duration was greater than 5 and 7 days and total IV antibiotic duration. Continuous antibiotic duration endpoints were assessed using Mann Whitney U and Fisher's Exact tests were used for categorical endpoints.

**Results:**

Of 480 patients screened, only 148 (31%) patients met early clinical stability criteria. The primary reason for not meeting criteria was requirement of supplemental oxygen on Day 3 or greater on antibiotics (n=145). This proportion is far lower than previous studies, including that by Dinh et al., which found 60-70% patients met stability criteria. Of the 90 patients included, 27 patients had a Tele-ID review. Patients with an intervention were less likely to receive > 5 days of antibiotics (59% vs 73%) and > 7 days of antibiotics (19% vs. 32%), although these differences were not statistically significant.

**Conclusion:**

This study relied on accuracy of oxygen charting, which may partly explain relatively lower proportion of patients meeting strict clinical stability criteria and likely limits feasibility of a scalable intervention. A pragmatically modified approach for this Tele-ASP intervention (i.e. allowance for no more than 1 unmet criterion) is an important area for further exploration and could improve the feasibility of a scalable telehealth intervention.

**Disclosures:**

**All Authors**: No reported disclosures

